# Failure of *E. coli *bacteria to induce preterm delivery in the rat

**DOI:** 10.1186/1477-5751-8-1

**Published:** 2009-01-04

**Authors:** Emmet Hirsch, Yana Filipovich, Roberto Romero

**Affiliations:** 1 Department of Obstetrics and Gynecology, NorthShore University HealthSystem, Evanston, IL, USA; 2 Department of Obstetrics and Gynecology, Feinberg School of Medicine, Northwestern University, Chicago, IL, USA; 3 Perinatology Research Branch, Eunice Kennedy Shriver National Institute of Child Health and Human Development, National Institutes of Health, Department of Health and Human Services, Bethesda, MD and Detroit, MI, USA

## Abstract

**Background:**

We sought to develop a model of bacterially induced preterm delivery in rats to parallel similar models in mice.

**Methods:**

Female Sprague-Dawley rats on day 17 of gestation (normal term = 21–22 days) were inoculated into the uterus with either 2 × 10^9 ^– 7 × 10^10 ^killed *E. coli *organisms, 1 – 4 × 10^8 ^live *E. coli *or sterile solution. These inoculations were made either via trans-cervical catheter or by direct intrauterine injection at laparotomy. Animals were then observed for delivery for variable periods up to term. Necropsies were performed and fetal viability was assessed.

**Results:**

No rats delivered prematurely after bacterial exposure (27 animals observed for at least 48 hours), and all animals followed to term (n = 3) delivered live pups. No dams exhibited signs of systemic illness. There was a statistically significant but small negative effect of killed *E. coli *on fetal viability (100% of 80 fetuses from 6 control pregnancies and 93% of 182 fetuses from 14 bacterially-treated pregnancies were alive at necropsy, p = 0.014). Live bacteria had a larger effect on fetal viability, with only 64% of 14 fetuses, 47% of 28 fetuses and 32% of 31 fetuses surviving after trans-cervical administration of 7 × 10^7^, 2 × 10^8 ^and 4 × 10^8 ^*E. coli*, respectively.

**Conclusion:**

Unlike mice, it has proven difficult to induce preterm labor in the rat using *E. coli *as a stimulating agent. The relevant literature is reviewed and hypotheses are offered to explain this phenomenon.

## Background

Preterm birth is the major cause of neonatal morbidity and mortality in the developed world [1]. Infection within the gestational compartment is thought to be the primary cause of a large proportion of cases of preterm labor, accounting for as many as 50% or more of premature deliveries, especially at very early gestational ages. Given the complexity inherent in the processes of parturition and the obstacles to conducting properly controlled and prospective studies in human subjects, animal models have proven helpful in developing an understanding of the physiology and pathophysiology of parturition [2]. Novel insights have been generated in animal models of infection-induced preterm labor in mice using a wide range of inflammatory stimuli from a large number of labs (small representative sample found in references [3-9]), including our own. Other well established models exist in rabbits [10-13] and non-human primates [14-16].

In order to capitalize on certain advantages afforded by the rat over the mouse (such as larger tissue samples and more easily conducted invasive monitoring), we sought to develop a model of infection-induced preterm labor in the rat. In this paper we report on the failure of this attempt, offer hypotheses for the causes of this failure and suggest possible directions for future study. It is notable that although there are abundant studies of rat parturition induced by progesterone antagonism [17-19] only two groups have reported preterm delivery with an infectious/inflammatory stimulus in rats in a total of three publications [20-22].

## Methods

### Rats

All procedures involving animals were approved by the Institutional Animal Care and Use Committee of NorthShore University HealthSystem. Timed pregnant Sprague-Dawley rats (254 – 380 gm) were obtained from Harlan (Indianapolis, IN) on day 15 of pregnancy and allowed to acclimate in the animal facility until the experiment was performed. In most cases and unless otherwise stated, experiments occurred on day 17 of a 21–22 day rat pregnancy.

### Bacterial preparation

Bacteria (a pathogenic strain of *E. coli *originally isolated from a patient with urosepsis and obtained from the American Type Culture Collection (ATCC #12014)) were stored in 50% glycerol at -80°C and processed as previously described [4,23]. Live and heat-killed bacteria from this strain have been used repeatedly by our group to induced preterm delivery in mice.

For experiments with killed bacteria, a small chip of the stock culture was thawed in Luria-Bertani (LB) medium, passaged 4 times prior to overnight culture in 4 L of medium and concentrated by centrifugation. The bacteria were then washed three times in phosphate-buffered saline (PBS) and resuspended in 8 ml of PBS, yielding a concentration of 1.7 × 10^11 ^bacteria per ml. This dense live bacterial prep was quantitated by plating serial dilutions for overnight culture just prior to the remaining stock being killed by boiling for 5 minutes. Non-viability of this boiled preparation was confirmed by overnight incubation of both plate and broth cultures. The stock of killed *E. coli *was aliquoted and frozen at -80°C. Prior to each experiment using killed bacteria, an aliquot was thawed and diluted in PBS to the desired concentration.

For experiments with live bacteria, *E. coli *were taken either from the frozen stock or from fresh plates and passaged twice, including one overnight culture in 3 ml of LB medium. At 10 AM, 100 μl of the overnight culture was added to 5 ml of sterile medium and grown at 37°C for 4 hours, when surgeries were performed. This bacterial prep was used undiluted and was kept at room temperature during surgery. The culture was quantified by plating before the first procedure and after the last procedure of the day, and the pre- and post-procedure counts were averaged to determine the inoculum used.

### Surgical procedures

Rats were anesthetized with a mixture of 10 mg/kg Ketamine and 1 mg/kg Xylazine intraperitoneally. They were then immobilized in the supine position with the external genitalia slightly overhanging the edge of the lab bench. A 3 mm lacrimal duct scope attached to an external light source was advanced into the vagina. Polyethylene tubing (Becton Dickinson #427416, I.D. 0.76 mm (.030") O.D. 1.22 mm (.048")) was joined to the barrel of the scope through a narrow channel created from strips of tape. The tubing could be advanced or withdrawn for cannulation of the cervix under direct visualization. The cannula (primed with injectate to remove air) was advanced for a distance of approximately 1 cm through the cervical opening prior to injection of 100 – 1000 μl of solution. Various infusion volumes were used to assess their potential differential effects. After an average of 30 seconds (to allow for fluid distribution through the uterine horn while minimizing egress through the cervix), the cannula was withdrawn and visualization continued for another 30 seconds to assess whether fluid leakage occurred. The rat was then returned to her cage and allowed to awaken from anesthesia.

Original attempts using both an external pressurized air supply and a hand-operated bulb to distend the vagina to ease visualization of the cervix were abandoned due to air entry into the uterine horns. Thereafter, we were easily able to visualize and catheterize the cervical orifice without any source of pressurized air.

In general, animals tolerated these procedures well. Four rats died within 20 hours of surgery (presumably from anesthetic complications, as no specific findings were seen on necropsy). An additional 2 animals developed a severe shock-like syndrome, again with no findings on necropsy. These 6 subjects were excluded from the analysis.

In some cases, direct intrauterine injection of bacteria was performed at laparotomy to assess the effects of route of administration. These abdominal procedures were fashioned after our existing mouse model [4,23]. A 2 cm midline incision was made in the lower abdomen and the right uterine horn was exposed. Bacteria or sterile medium were then injected in volumes of 100 – 1000 μl into the midsection of the right uterine horn at a site between two adjacent fetuses. The peritoneal cavity was then closed with interrupted sutures and the skin was closed with staples.

Surgical procedures lasted 10 – 15 minutes. Animals recovered in individual, clean cages in the animal facility and underwent twice-daily observations. The number of live-born or dead pups was noted. Preterm delivery was defined as the finding of at least one fetus in the cage or in the lower vagina within 48 hours of surgery.

In some cases, early euthanasia and necropsy were performed to assess catheter placement, dye distribution or fetal viability. Fetal viability was assessed by color, spontaneous movement and visible vascular pulsations in either the chest cavity or the vessels within fetal membranes. Other animals were observed for various periods of time through one week after delivery to record other observations, such as time to delivery and viability of delivered pups. Animals were euthanized by CO_2 _inhalation plus thoracotomy.

### Statistics

Statistical analysis of categorical values was by chi-square or contingency tables, with Fisher exact correction as needed.

## Results

### Development of a trans-cervical uterine infusion model in the rat

A total of 9 rats on days 14–18 of pregnancy underwent catheterization and immediate euthanasia to assess catheter position. A separate group of 14 pregnant rats on gestational days 17–19 (7 rats on day 17, 6 rats on day 18 and 1 rat on day 19) was instilled with PBS with or without sterile bromphenol blue dye in a total volume of 100–500 μl and observed for up to 70 hours to characterize the distribution of dye and any fetal effects.

After an initial learning period, we were able consistently to catheterize one uterine horn to the desired distance of 1 cm and infuse it with the dye solution with retention of dye in the uterine horn, continued viability of fetuses (verified by both immediate and delayed necropsy) and minimal leakage out the cervix and vagina. No control animals delivered within 48 hours, the previously determined cutoff for preterm delivery (n = 14), and 11 of 12 rats followed to term delivered healthy litters (averaging 11.1 ± 2.7 pups per litter). The 12th rat had vaginal bleeding 20 hours after surgery and upon necropsy was found to have 6 absorbing conceptuses in one uterine horn along with 10 empty implantation sites distributed in both horns, suggestive of prior delivery from these sites. Therefore, we concluded that the trans-cervical infusion of various volumes of sterile solution was feasible and rarely affected the course of pregnancy.

### Trans-cervical intrauterine infusion of a pathogenic strain of killed E. coli fails to induce preterm delivery

The trans-cervical infusion method was used to instill 2 × 10^9 ^– 8 × 10^10 ^killed *E. coli *organisms into the uteri of 14 pregnant rats at 17 days of gestation (Table 1). None of these animals delivered within 48 hours. Among these pregnancies, 6 were euthanized and necropsied at 48 hours to assess fetal status (see results below). The remaining 8 rats were observed for 70 hours without any preterm deliveries. No animals exhibited signs of systemic illness (such as piloerection, decreased mobility or decreased feces production).

**Table 1:** Effect of intrauterine *E. coli *inoculation on preterm delivery.

Treatment	Number of *E. coli *organisms	Number of animals	Preterm delivery rate (%)
Trans-cervical infusion			
Control	N/A	16	0
Killed *E. coli*	0.2 – 8 × 10^10^	14	0
Live *E. coli*	1 – 4 × 10^8^	8	0
Infusion at laparotomy			
Control	N/A	1	0
Live *E. coli*	2–7 × 10^8^	5	0

'Preterm' was defined as 48 hours after surgery, however some animals were observed for longer periods, up to term (see text).

### Intrauterine infusion of live E. coli fails to induce preterm delivery

Our existing mouse model is characterized by a dramatic difference in potency of killed versus live bacteria for induction of preterm delivery (live bacteria are more potent by 5–6 orders of magnitude, with as few as 2000 organisms leading to preterm delivery) [4]. Therefore, we also experimented with live bacteria in an attempt to maximize the potential for inducing preterm delivery in rats. Eight pregnant rats were inoculated trans-cervically with 1 – 4 × 10^8 ^live *E. coli *organisms. None of these animals delivered prematurely, including 3 animals that were followed to term, each of whom delivered live pups. None of the rats treated with live bacteria showed signs of systemic illness.

Finally, in order to investigate the effect of method of inoculation, 5 pregnant females were inoculated with 2 – 4 × 10^8 ^live *E. coli *organisms by direct injection into the right uterine horn at laparotomy. None of these 5 rats delivered prematurely.

### Fetal effects of intrauterine bacterial infusion

The effect of intrauterine bacterial infusion on fetal viability was assessed by timed necropsy after surgery. One hundred percent of 80 fetuses from 6 mothers who received control (non-bacterial) injections and were euthanized 24 – 70 hours later were still alive at the time of necropsy. Also, as noted above, dams followed to term after control injections delivered large, healthy litters. In 14 litters treated with 2 × 10^9 ^– 8 × 10^10 ^killed bacteria, 93% of 182 fetuses were alive at necropsy 48 – 70 hours later (p = 0.014, comparing control to killed bacterial injection). There were no significant trends associating inoculum size with fetal survival. Thus, killed *E. coli *has a statistically significant though small impact on fetal viability.

**Figure 1:**
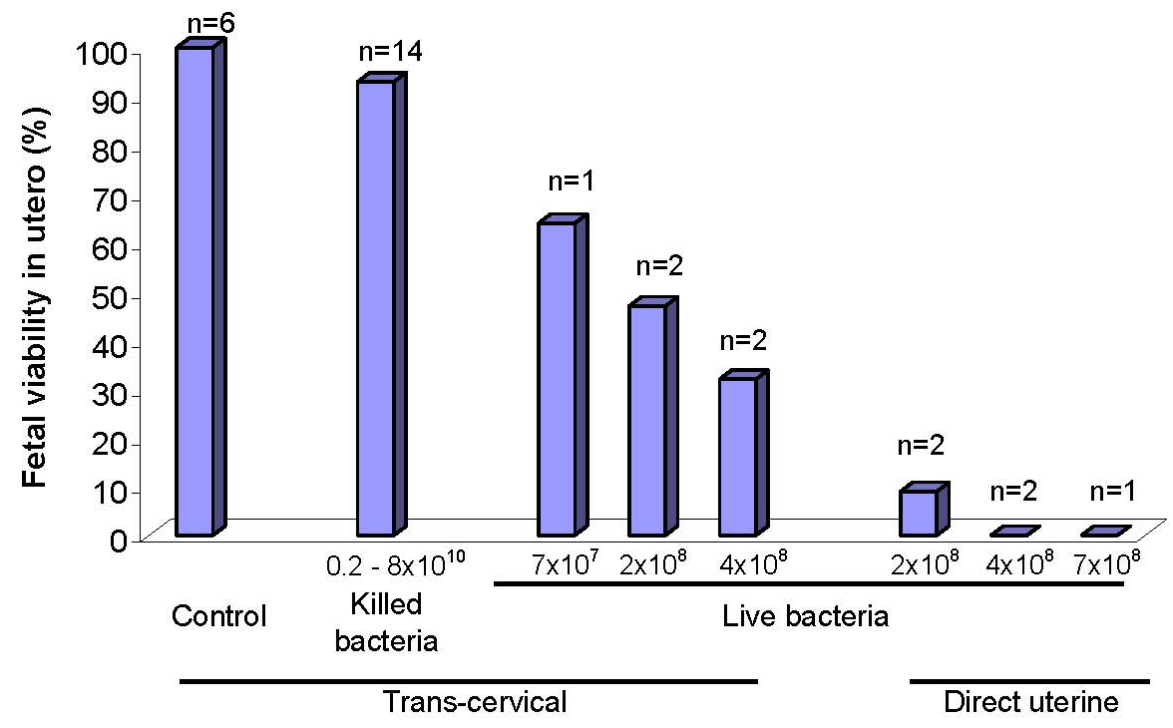
**Effect of intrauterine treatment on fetal viability**. Female rats on day 17 of pregnancy were inoculated with either sterile solution (control), killed *E. coli *or live *E. coli *bacteria, by either trans-cervical infusion or direct intrauterine injection at laparotomy. Animals were euthanized 24–70 hours later and fetal viability was determined. Displayed on the Y-axis is the percentage of fetuses alive at the time of autopsy. The number of treated dams in each group is also displayed.

In contrast, live bacteria had a large effect on fetal viability that was dependent upon route of administration. Trans-cervical inoculation with live *E. coli *was associated with survival at necropsy 48 hours later in 64% of 14 fetuses, 47% of 28 fetuses and 32% of 31 fetuses from 5 pregnant rats administered 7 × 10^7^, 2 × 10^8 ^or 4 × 10^8 ^live *E. coli*, respectively (p = 0.13) (Figure 1). Direct administration of live bacteria by intrauterine injection at laparotomy produced even more profound effects on fetal survival, with only 3.4% of 63 fetuses from 5 pregnant dams still alive at necropsy 48 hours later (p = 0.0006 for comparison of the trans-cervical and direct injection routes of administration of 2 × 10^8 ^bacteria and p = 0.003 for comparison of the two routes of administration of 4 × 10^8 ^bacteria). Finally, each of 3 pregnant dams who received 1 – 2 × 10^8 ^live *E. coli *trans-cervically and were followed through spontaneous delivery produced live pups at term, but their number was diminished (average 3 per litter), compared with 10.2 pups per litter in control animals (p = 0.001).

## Discussion

The present study describes the inability to induce preterm delivery in pregnant rats using live or killed *E. coli*. This experience stands in sharp contrast to published experience with mice, in which live or killed *E. coli *as well as multiple other inflammatory/infectious stimuli produced preterm delivery [3,4,6-9,24,25]. These stimuli include a variety of bacteria, pathogen extracts (e.g. LPS, peptidoglycan, lipoteichoic acid, poly inosinic:cytidylic acid), cytokines (e.g. IL-1, TNF), and others. The strain of *E. coli *used in the present study has been used successfully in models of preterm birth not only by our group but by others also in mice [8,26] and rabbits [12,27].

We could identify in the literature only two laboratories that reported induction of preterm delivery using an infectious/inflammatory stimulus in rats [20-22]. In work from the first group, Sprague Dawley rats underwent intrauterine implantation of catheters on day 15 or 16 followed on day 17 of pregnancy by infusion of 25 μg or 50 μg of LPS over 4 hours. Saline treatment was followed by delivery in an average of 117 hours, whereas 25 μg and 50 μg of LPS produced delivery in 82 and 63 hours, respectively, together with increased production of prostaglandin F2α metabolite in placentas of LPS-exposed animals [20]. In a separate study from the same group, a 50 μg infusion of LPS produced preterm delivery in an average of 92 hours, which was approximately one day before delivery in saline-treated rats [22]. Treatment with IL-10 restored delivery to term. It is not clear why there was a discrepancy of nearly 30 hours in the interval to delivery between these two studies in rats treated with 50 μg LPS. In a comment, the authors note that slow infusion over 4 hours appears to be important for 'optimal results' [20]. For our studies we chose 48 hours as a reasonable cut-off for 'preterm delivery' to avoid approaching term and because this cutoff is practical in the mouse (in which delivery in similar models usually occurs in less than 24 hours). When delivery did not occur in this time frame, we extended our observations to term and still did not observe early delivery.

In work from the second group, Wistar rats were treated with 25 μg/kg of *E. coli *LPS intraperitoneally on day 16 of pregnancy [21]. Although no animals in this study were treated with control injections, the average time to delivery was approximately 22 hours. It should be noted that assuming an average weight of 333 gm per rat, these subjects would have received 8.3 μg of LPS each. Somewhat surprisingly, intraperitoneal treatment with erythromycin significantly prolonged the interval to delivery, by up to 50 hours.

Whether the different potencies reflected in the above two experimental designs is due to route of administration (intrauterine versus intraperitoneal), rate of infusion, rat strain (Sprague-Dawley versus Wistar) or some other factor is not clear. In the present report, Sprague Dawley rats treated with 2 × 10^9 ^– 8 × 10^10 ^killed *E. coli *would have received between 20 μg and 800 μg of LPS each, assuming that each *E. coli *bacterial cell contains 9.7 × 10^-15 ^gm LPS [28].

Far more numerous than the 3 studies cited above that demonstrate preterm birth after LPS administration are the studies that use hormonal manipulation (primarily progesterone antagonism) to induce preterm delivery in rats (examples to be found in [18,29,30]). This notable fact is reinforced by another, namely that the literature contains several examples of experimental LPS exposure in rats after which observation periods of sufficient length were conducted to effectively rule out subsequent preterm delivery. For example, *E. coli*-derived LPS (400 μg/kg intraperitoneally) induced cervical changes in Sprague Dawley rats when administered on day 14 of pregnancy [30]. Animals were harvested 2 days after treatment with no cases of preterm delivery reported. In a study of the effect of prenatal LPS on neurobehavioral development, pregnant Wistar rats were administered 2 mg/kg of *E. coli *LPS subcutaneously on each day of pregnancy from conception through delivery [31]. Though maternal LPS treatment induced significant changes in neonatal neurobehavioral outcomes as well as dopaminergic neurotransmission and synaptophysin expression, there was no reported effect on timing of delivery. A different study in which Sprague Dawley rats were treated with LPS (100 μg/kg intraperitoneally) every day from day 14 to day 20 of pregnancy demonstrated a significant increase in fetal death and reduced size of surviving fetuses compared with controls [32]. Within the uteri of treated dams there was increased apoptosis, a doubling of TNFα levels and a tripling of nitric oxide and myeloperoxidase levels, however no preterm labor was reported. In a study focusing on fetal/neonatal brain injury, *E. coli *LPS treatment (500 μg/kg intraperitoneally) of pregnant Sprague Dawley rats on day 18 and again on day 19 of pregnancy produced white matter injury, myelination defects and apoptosis and increased expression of IL-1β, TNFα and IL-6 in pup brains 7 days after birth [33]. Again, no preterm delivery was reported. In a Chinese language publication available in English in abstract form, treatment of Sprague Dawley rats with intraamniotic LPS (quantity not provided) on day 15 of pregnancy produced both histologic changes and reduction in expression of vascular endothelial growth factor (VEGF) and its receptors (Flk-1 and Flt-1) in neonatal lungs, but animals delivered at term [34].

Why has the induction of inflammation-induced preterm birth been so difficult and variable in the rat, while it is so readily accomplished in mice and other species? The results cited above and the fact that in the present study there were measurable effects on fetal survival suggest that the pregnant Sprague Dawley strain of rat is responsive to *E. coli*. Among the demonstrable effects of LPS in pregnant rats are uterine apoptosis and increased expression of TNFα, nitric oxide and myeloperoxidase [32], increased prostaglandins in the amniotic fluid and an increase in *in vitro *myometrial contractile activity in response to oxytocin [35], and an increase in both spontaneous and agonist-induced uterine contractions in myometrial strips isolated from LPS-treated rats [36]. Therefore, it seems likely that failure to induce preterm delivery in this model of bacterial infection is specific for parturition, rather than due to a global hyporesponsiveness to either Gram negative bacteria or LPS.

Among the possible explanations for this phenomenon is that LPS may have a differential effect among different rodent species with respect to parturition-related factors. A number of genes have been identified in the mouse that are critical for a normal response in parturition models. It is possible that some of these genes or others not yet identified are differentially expressed in various rat strains, thus accounting for a lack of responsiveness. Further comparative analysis of genomic sequence information (particularly between mice and rats) may provide valuable insights in this regard. Terrone noted that high doses of LPS induced intrauterine fetal demise, while lower doses tended to produce fewer fetal deaths and preterm delivery [22]. Two groups have reported that LPS increases uterine myometrial contractility in rat uterus in response to oxytocin [35,36]. However, spontaneous uterine contractions induced by LPS in the rat do not appear to be mediated by prostaglandins, as prostaglandin inhibitors fail to diminish this response [36]. This appears contrary to relevant mechanisms in other species. Finally, uterine contractions in the rat may be dissociated from cervical ripening. In one study, a platelet-activating factor (PAF-) antagonist blocked cervical changes induced by LPS but not those induced by the antiprogestin RU-486 [30]. Perhaps the rat laboring after exposure to LPS or *E. coli*, unlike the spontaneously laboring rat at term, does not experience the kind of cervical modifications ('ripening') that allow it to deliver.

We observed a difference in the fetal toxicity of a live bacterial inoculum between trans-cervical inoculation with a catheter and direct intrauterine inoculation at laparotomy. We suspect that this difference may be due to delayed leakage of the trans-cervical inoculum through the dilated cervical orifice left behind by the catheterization procedure, thus effectively diminishing the size of the inoculum.

It is our hope that publication of the present negative results will either spur successful efforts to create an improved rat model or lead to potentially useful insights as to why this rodent behaves so differently from the mouse. Such insights have the potential to lead to applications in humans that will diminish the burden of premature birth.

## Competing interests

The authors declare that they have no competing interests.

## Authors' contributions

EH contributed to the conception and design, development of the rat model, analysis of data, and drafting of the manuscript. YF contributed to the development of the rat model, carried out the experiments, analyzed results and helped draft the manuscript. RR contributed to the conception and design, analysis of data, and drafting of the manuscript. All authors read and approved the final manuscript.
